# Substituting hospital-based outpatient cardiology care: The impact on quality, health and costs

**DOI:** 10.1371/journal.pone.0217923

**Published:** 2019-05-31

**Authors:** Tessa C. C. Quanjel, Marieke D. Spreeuwenberg, Jeroen N. Struijs, Caroline A. Baan, Dirk Ruwaard

**Affiliations:** 1 Department of Health Services Research, Care and Public Health Research Institute, Faculty of Health Medicine and Life Sciences, Maastricht University, Maastricht, the Netherlands; 2 Research Centre for Technology in Care, Zuyd University of Applied Sciences, Heerlen, the Netherlands; 3 Department for Quality of Care and Health Economics, Centre for Nutrition, Prevention and Health Services, National Institute for Public Health and the Environment, Bilthoven, the Netherlands; 4 Department for Public Health and Primary Care, Leiden University Medical Centre, Leiden, the Netherlands; 5 Scientific Centre for Transformation in Care and Welfare (Tranzo), University of Tilburg, Tilburg, the Netherlands; Chinese Academy of Medical Sciences and Peking Union Medical College, CHINA

## Abstract

**Background:**

Many Western countries face the challenge of providing high-quality care while keeping the healthcare system accessible and affordable. In an attempt to deal with this challenge a new healthcare delivery model called primary care plus (PC+) was introduced in the Netherlands. Within the PC+ model, medical specialists perform consultations in a primary care setting. PC+ aims to support the general practitioners in gatekeeping and prevent unnecessary referrals to hospital care. The aim of this study was to examine the effects of a cardiology PC+ intervention on the Triple Aim outcomes, which were operationalized by patient-perceived quality of care, health-related quality of life (HRQoL) outcomes, and healthcare costs per patient.

**Methods:**

This is a quantitative study with a longitudinal observational design. The study population consisted of patients, with non-acute and low-complexity cardiology-related health complaints, who were referred to the PC+ centre (intervention group) or hospital-based outpatient care (control group; care-as-usual). Patient-perceived quality of care and HRQoL (EQ-5D-5L, EQ-VAS and SF-12) were measured through questionnaires at three different time points. Healthcare costs per patient were obtained from administrative healthcare data and patients were followed for nine months. Chi-square tests, independent t-tests and multilevel linear models were used to analyse the data.

**Results:**

The patient-perceived quality of care was significantly higher within the intervention group for 26 out of 27 items. HRQoL outcomes did significantly increase in both groups (P <0.05) but there was no significant interaction between group and time. At baseline and also at three, six and nine months’ follow-up the healthcare costs per patient were significantly lower for patients in the intervention group (P<0.001).

**Conclusions:**

While this study showed no improvements on HRQoL outcomes, PC+ seemed to be promising as it results in improved quality of care as experienced by patients and lower healthcare costs per patient.

## Introduction

Many Western countries face the challenge of providing high-quality care while keeping the healthcare system accessible and affordable [1, 2]. In an attempt to deal with the challenge of realizing sustainable and high-quality healthcare systems, newly introduced initiatives should focus on simultaneously pursuing three aims: improving the health of the population and quality of care as experienced by patients, and at the same time reducing the increase of healthcare costs, known as the Triple Aim [3].

The World Health Organization (WHO) encourages countries to orient their healthcare system towards a strengthened primary health care [4, 5]. WHO assumes that better use of primary care services is associated with reduced healthcare costs and higher patient satisfaction [6]. Moreover, strong primary care systems are associated with positive effects such as better health outcomes, lower rates of unnecessary hospitalizations, and relatively lower socioeconomic inequality [7–9]. However, Kringos and colleagues also found that total healthcare expenditures were higher in countries with a stronger primary care structure [7]. Overall, the evidence supports that strengthening primary care systems results in improved (healthcare system) outcomes, however further research is recommended.

Redesigning healthcare delivery models to limit the growth of healthcare costs and to increase quality of care is high on the political agenda in the Netherlands. Expenditure on specialized care increased by more than 55% during the past decade to more than 27 billion euros in 2016 [10]. In 2013, the Dutch Ministry of Health, Welfare and Sport, the healthcare providers and the insurers agreed to shift (less complex) care from the hospital towards the primary care setting [11, 12]. Following this agreement, stakeholders in several regions started experimenting with a new healthcare delivery model called Primary Care Plus (PC+). This new care delivery model PC+ aims to facilitate the substitution of care and prevent unnecessary referrals to (outpatient) hospital care by enhancing communication and collaboration between medical specialists and general practitioners (GPs). The Dutch GPs act as strict gatekeepers of the healthcare system and hospital- and specialist care are only accessible through GP referral [13]. Moreover, within the PC+ model, medical specialists perform consultations in the primary care setting to support GPs and strengthen the gatekeeping system [14, 15].

Internationally comparable models such as PC+ are specialist outreach services, shifted outpatient clinics and joint consultations [16–21]. Previous research has shown that these models could result in improved patients’ satisfaction, shorter waiting times, improved access to specialist care, fewer diagnostic tests and reduced referrals to hospital care [16–18, 20, 21]. On the contrary, some authors point out that relocation of specialist care from the secondary to the primary care setting may increase healthcare costs [16–19]. Previous research on PC+ emphasized the importance of investigating which patients are (less) suited to PC+. To achieve efficiency, PC+ interventions should exclude patients who need hospital care anyway and PC+ should be provided in a neutral environment (such as an independent PC+ centre) instead of providing specialist consultations in GP practices [15, 22]. An independent PC+ centre enhances the ability to use specialist time efficiently and avoid overuse of care because of the close working relations between GPs and specialists [15]. Although many interventions focused on shifting specialist care from the hospital setting towards the primary care setting have been evaluated, evidence is still inconsistent. In particular, in relation to this relatively new healthcare delivery model PC+, evidence of its impact is lacking.

This study evaluates a cardiology PC+ centre where cardiologists provide consultations for non-acute and low-complexity patients with cardiology-related complaints in a primary care setting [23]. PC+ is intended to achieve improvements on the Triple Aim [3]. Therefore, this study aims to examine the effects of this cardiology PC+ centre on the Triple Aim outcomes. More specifically, this study focuses on quality of care as experienced by patients, health of the population measured with health-related quality of life (HRQoL) outcomes, and healthcare costs per patient (operationalized by healthcare spending per patient). Overall, this current paper aims to answer three questions:

- Is the patient-perceived quality of care higher in PC+ compared to care-as-usual (i.e. hospital-based outpatient care)?
- Does PC+ result in improved or at least equal HRQoL outcomes compared to care-as-usual (i.e. hospital-based outpatient care)?
- Does PC+ result in lower healthcare costs per patient (operationalized by healthcare spending per patient)?

## Methods

### Study design

This is a practice-based quantitative study with a longitudinal observational design based on patient questionnaires and administrative healthcare data. Patient questionnaires were used to examine quality of care as experienced by patients and HRQoL outcomes. Administrative healthcare data of the cardiology PC+ centre and the affiliated hospital were obtained to investigate the healthcare costs. The study is approved by the Medical Research and Ethics Committee of the Maastricht University Medical Centre (METC 15-4-032). A detailed description of the setting, intervention and study population is described in a study protocol [23].

### Setting and the intervention

The cardiology PC+ centre, which commenced in October 2014, is located in the most southern part of the Netherlands. The PC+ centre is an initiative of the regional care group (i.e. a legal entity formed by all GPs in the region), the regional hospital, the patient representative foundation and the dominant health insurance company in the region. The region consists of 270 000 residents, approximately 135 GPs and one hospital. The population is characterized as relatively old, unhealthy and with a low socio-economic status as compared to the overall Dutch population [24].

The intervention is a PC+ centre that provides relocated specialist care, i.e. outpatient cardiology care is shifted from the hospital setting towards the primary care setting. GPs can refer patients to the PC+ centre where cardiologists perform consultations in a primary care setting. Hospital diagnostic tools are available including an electrocardiogram (ECG), an ergometer and an ultrasound device. The appointment at the PC+ centre consists of diagnostic tests and a consultation with the cardiologist. The cardiologist explains the results of the diagnostic tests and, afterwards, sends these results with the diagnosis and recommendation for further treatment (if needed) to the GP. The GP remains clinically responsible for the patient and the GP discusses the further treatment with the patient. The two overall recommendations for further treatment are: 1) the patient may remain in the primary care setting; 2) the patient needs to be referred to specialist care in the hospital setting.

### The study population

The study population consists of adult patients (≥18 years) with non-acute and low-complexity cardiology-related health complaints, and the referral consists of one of the following indications: heart palpitations, heart murmur, cardiac screening, suspected arrhythmia, atypical chest pain, reduced exercise capacity, collapse, abnormal ECG, dyspnoea, suspected heart failure, suspected coronary sclerosis, analyses of atrial fibrillation, stable Angina Pectoris [22]. Patients who were already diagnosed with cardiology-related health problems by a cardiologist and patients with acute health problems who require immediate hospital care and/or patients arriving at the emergency department of the hospital were excluded from participation in this study.

The allocation of patients was not random. The GPs were instructed about the in- and exclusion criteria of PC+ and based on their clinical expertise, and shared-decision making, they decided whether a patient was referred to the PC+ centre. The researchers did not have any influence on the referral decision. The intervention group consisted of all patients referred to the cardiology PC+ centre.

During the starting phase of this PC+ centre, the GP did not refer all eligible patients to PC+. Consequently, the first two years after the start of the PC+ centre, there were still patients referred to the hospital-based outpatient care (from here reported as ‘HBOC’), who could also have been referred to PC+ according to the in- and exclusion criteria. HBOC is provided in the general hospital located in the same region as the PC+ centre. HBOC is considered as care-as-usual. Patients referred to HBOC received the same diagnostic tests as within the PC+ centre as well as a consultation with a cardiologist. The control group consisted of patients referred to HBOC, who could also have been referred to PC+. The coordinator of the cardiology department in the hospital decided whether a patient was eligible for the control group, based upon the in- and exclusion of this study.

### Data collection

The study was based on two types of data sources: patient questionnaires and administrative healthcare data. The data collection took place according to two separate tracks.

#### Patients’ experience of care and health-related quality of life (HRQoL)

**Fig 1:**
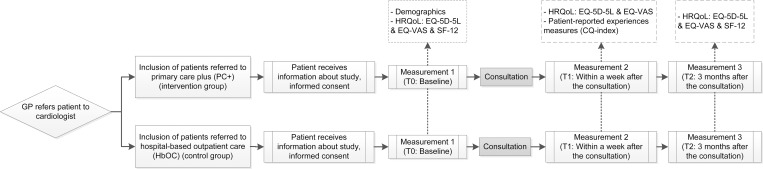
Patient flow and patient questionnaire measurements: Patients’ experiences of care and HRQoL outcomes.

The data collection regarding the patients’ experience of care and the HRQoL was based on questionnaires. For the patient questionnaire study, the patient enrolment of both the intervention group and the control group started when a GP referred a patient to PC+ or HBOC. All eligible patients were asked to participate in the study. Before the consultation at the PC+ centre or the hospital, the patients received an information letter with an informed consent form. In this letter, they were asked if they would like to participate in the study. The flow of participants and the data collection measurements regarding patients’ experience of care and HRQOL are summarized in Fig 1. All participants signed an informed consent and self-reported questionnaires were administered to all patients at baseline (T0), within a week after consultation (T1) and at three-month follow-up (T2). The inclusion of patients started in April 2015 and the last follow-up measurements were carried out in October 2017. Based on a sample size calculation it was necessary to include 358 patients per group, when assuming a power of 80% and a significance level of 0.05 [23]. Patients’ experience of care was measured within a week after consultation (T1) with 27 items of the Consumer Quality (CQ) index (Summary of Questions in S1 File). The CQ index is a standardized method for measuring experiences of patients with healthcare [25]. HRQoL was measured using two generic health status questionnaires: the EuroQol five-dimensional questionnaire with five levels (EQ-5D-5L) including the EuroQol Visual Analogue Scale (EQ-VAS) and the Short-Form Health Survey (SF-12). The EQ-5D-5L was included in all three measurements (T0, T1 and T2). The SF-12 was included in the baseline measurement (T0) and after three-month follow-up (T2). HRQoL instruments assess the effectiveness of a service in relieving symptoms or changing health status in ways that patients’ value [26]. The EQ-5D-5L consists of five questions corresponding to the dimensions mobility, self-care, usual activities, pain/discomfort and anxiety/depression [27]. The scores range from -0.446 to 1 (worst- to best imaginable health status), using the Dutch utility tariff [28]. The EQ-VAS is a single question about self-rated overall health, with scores ranging from *0* to *100* (worst- to best imaginable health state). The SF-12 consists of 12 questions measuring the physical and mental health by means of two summary scores; a physical component summary (PCS) and a mental component summary (MCS) [29].

#### Healthcare costs

**Fig 2:**
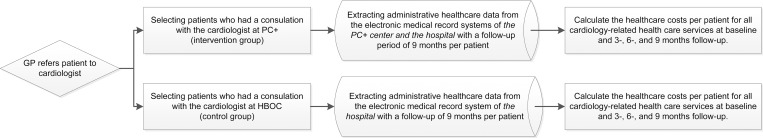
Collecting administrative healthcare data to measure the healthcare costs per patient.

The evaluation of the healthcare costs was based on administrative healthcare data at patient level extracted from the electronic medical record systems (Fig 2). All patients who met the inclusion criteria of the study population and were referred by their GP to the cardiologist at the PC+ centre (intervention group) or at the HBOC (control group) between 1 January and 31 December in 2016 were included. Each patient was followed for nine months, meaning that data were gathered until October 2017. The administrative healthcare data of these patients were retrospectively selected from the electronic medical record systems of the PC+ and hospital. For each patient all healthcare data concerning cardiology-related healthcare services provided in the PC+ centre and the hospital were extracted from the electronic medical record system. The data consisted of all cardiology-related healthcare services provided at the PC+ centre and the hospital including outpatient as well as inpatient care (e.g. diagnostic tests, consultations, surgeries). Healthcare costs related to primary care, social care and drugs are not included in this study. The costs for all cardiology-related healthcare services were estimated using the national database of the Dutch Healthcare Authority (*Nederlandse Zorgautoriteit (NZa)*) [30]. In the Netherlands, hospital services are paid through a diagnosis-related-group (DRG) type of system called Diagnosis Treatment Combinations (*Diagnose Behandel Combinaties*, DBCs) [13]. The administrative healthcare data included all recorded DBCs at patient level. The national database with standardized average costs of DBCs was used to compute the cardiology-related healthcare costs per patient [30]. The healthcare costs related to the PC+ consultation consisted of a fixed rate per patient; labelled as baseline costs within the intervention group. The baseline costs in the control group were determined by the first recorded DBC. In addition, the PC+ centre, the hospital and insurer agreed that if a patient is referred to hospital care after a consultation in PC+ centre, the costs for PC+ should be integrated in the first recorded DBC opened in the hospital. Because the data received still consisted of both the costs for PC+ and the (total) costs for the first DBC, the healthcare costs for PC+ were subtracted from the first DBC opened in the hospital.

### Data analysis

Descriptive statistics were computed to provide information about the study population. Data were described using absolute counts and percentages for categorical variables, and means and standard deviation for continuous variables. Additionally, statistical model assumptions were examined before conducting further analyses. First, the majority of the items related to patients’ experience of care were dichotomized before analysing, summarized as e.g. ‘Satisfied’ versus ‘Unsatisfied’ or ‘Yes’ versus ‘No’. Afterwards, these categorical items were analysed using Pearson Chi-square tests; counts, percentages and *p*-values were reported. Additionally, continuous items were analysed using independent t-tests; 95% confidence intervals (CIs) and *P*-values were reported. Second, HRQoL outcome measures (i.e. EQ-5D-5L, EQ-VAS, SF-12 PCS and SF-12 MCS) and the healthcare costs were analysed using multilevel linear modelling (MLM); estimates, standard errors (SE) and 95% confidence intervals (CI) are reported. MLM was chosen because the observations at different time points are dependent (i.e. correlated data). All MLM in the present study consist of two levels (time and participants). More specifically, within the MLM the gain score analysis (GSA) technique was chosen to analyse the data. In GSA, the group effect is adjusted for the complete group differences in the baseline measurement [31, 32]. Furthermore, potentially significant confounding variables (gender and age) were entered into the models. If a variable changed the effect by 10% or more, it was considered as a confounder. All analyses were performed using IBM SPSS Statistics, version 24, and *p*-values <0.05 were considered as significant.

## Results

### Patients’ experience of care and health-related quality of life (HRQoL)

Within the PC+ group, 429 patients filled in the first questionnaire (T0: response rate 23.4%). The second questionnaire was filled in by 370 patients (T1: response rate 20.2%), and 327 patients filled in the third questionnaire (T2: response rate 17.8%). In the control group, 321 patients filled in the first questionnaire (T0: response rate 10.7%. The second questionnaire was filled in by 291 patients (T1: response rate: 9.7%) followed by 261 patients who also filled in the third questionnaire (T2: response rate 8.7%). The mean age of patients in the intervention group was 57.3 years (SD±12.9), which was lower compared to 63.9 years (SD±13.2) in the control group (*p* <0.000). Additional information on patient characteristics and scores on each outcome measurement are presented in S1 and S2 Tables.

The results regarding the patients’ experience of care are presented in Table 1. The intervention group scored significantly higher, indicating a significant favourable outcome, on all items (p <0.05), with the exception of ‘*findability of the location*’. Table 1 is subdivided into four parts. Firstly, 16 items about the general experience of care. For example, it shows that 98.1% of the patients in the intervention group were satisfied with the waiting time for the appointment, as compared to 85.1% of the patients in the control group (*p* <0.000). Secondly, five items specifically focused on the experience with the medical specialist, i.e. the cardiologist. For example, as shown in Table 1, 99.5% of the patients were satisfied with the time the medical specialist spent with them during the consultation in the PC+ centre, compared to 93.1% of the patients in the control group (*p* <0.000). Thirdly, four additional items related to the patients’ experience of care are given, e.g. it indicates that 83.5% of the patients referred to PC+ had an appointment within 7 days, as compared to 36.1% of the patients in the control group (*p* <0.000). The last part of Table 1 presents the grades given by the patients, e.g. the PC+ centre got a favourable mean grade of 9.04 (SD±0.95) compared to a mean of 8.05 (SD±1.14) given by the patients who visited the hospital (*p* <0.000).

**Table 1:** Results for patients’ experience of care.

**General items related to the experience of care** ^**A**^			
	**Intervention group (PC+) (N = 370)**	**Control group** **(HBOC) (N = 291)**	**P-value** ^**B**^
	Satisfied % (n)^C^	Satisfied % (n)^C^	
Satisfaction with waiting time for appointment*	98.1% (362)	85.1% (245)	<0.000
Findability of location	98.4% (364)	99.0% (286)	0.522
Feeling welcome and comfortable*	99.5% (368)	91.4% (265)	<0.000
Helpful healthcare assistant*	100.0% (370)	97.3% (283)	0.001
Understandable explanation by healthcare assistant*	99.7% (369)	95.5% (274)	<0.000
Sufficient facilities in waiting room*	98.6% (365)	82.5% (236)	<0.000
Healthcare professionals were informed about the complaint*	95.7% (353)	86.0% (246)	<0.000
Complaint was taken seriously*	99.5% (368)	95.1% (274)	<0.000
Healthcare professionals listened carefully*	98.9% (365)	94.8% (275)	0.002
Healthcare professionals spent enough time*	99.5% (368)	95.5% (277)	0.001
Healthcare professionals treated you with respect*	100.0% (369)	95.5% (274)	<0.000
Competence of healthcare professionals*	100.0% (370)	98.6% (278)	0.022
Overall help of healthcare professionals*	100.0% (370)	95.1% (274)	<0.000
Understandable explanation of healthcare professionals*	99.7% (368)	94.1% (271)	<0.000
Opportunity to ask questions*	98.4% (364)	93.8% (270)	0.002
Collaboration and alignment with GP*	98.1% (355)	87.5% (251)	<0.000
**Specific items related to the experience of care with the medical specialist (i.e. the cardiologist)**			
	**Intervention group** **(N = 370)**	**Control group** **(N = 291)**	**P-value**^**B**^
**The medical specialist …**	Satisfied % (n)^C^	Satisfied % (n)^C^	
… took enough time*	99.5% (365)	93.1% (270)	<0.000
… was informed about complaint*	95.9% (352)	91.3% (262)	0.014
. . . explained the results of the consultation and diagnostics sufficiently and in an understandable way*	99.2% (362)	94.1% (271)	<0.000
… provided information about treatment options*	95.5% (340)	90.4% (253)	0.010
… involved the patient in decision about the treatment*	89.0% (154)	78.4% (127)	0.008
**Additional items related to the patients’ experience of care**			
	**Intervention group** **(N = 370)**	**Control group** **(N = 291)**	**P-value** ^**B**^
	Yes % (n)^C^	Yes % (n)^C^	
Waiting time for appointment less than 8 days*	83.5% (308)	36.1% (103)	<0.000
Waiting time in waiting room less than 30 minutes*	92.7% (342)	87.1% (250)	0.017
Recommend PC+ centre/ hospital to family and friends*	99.5% (367)	92.0% (266)	<0.000
Recommend medical specialist to family and friends*	98.1% (358)	89.0% (252)	<0.000
**Grades**			
	**Intervention group** **mean (±SD) (N = 370)**	**Control group** **mean (±SD) (N = 291)**	**P-value** ^**B**^
Grade for PC+ centre / HBOC (0–10)*	9.04 (±0.95)	8.05 (±1.14)	<0.000 ^D^
Grade for medical specialist (0–10)*	8.91 (±1.06)	8.27 (±1.33)	<0.000 ^E^

Notes

* Item on which the groups differ significantly with a p-value < 0.05

^A^ All Healthcare professionals involved, including doctors’ assistants and nurses at the PC+ centre/ hospital

^B^ A chi-square test was used to test whether the two groups differ significantly

^C^ The n represents the number of responses on the particular item, missing values are excluded (the N represents the response on the questionnaire)

^D^ 95% CI = -1.143 –-0.824;

^E^ 95% CI = -0.828 –-0.451.

Regarding the EQ-5D-5L, EQ-VAS and SF-12 MCS models, the results showed that age was significantly associated with the HRQoL; an increase in age was significantly associated with a lower HRQoL. Additionally, it was found that age was a possible confounder within the EQ-5D-5L, the EQ-VAS and SF12 MCS models; when age was added to the model, the effect changed by more than 10%. Interaction terms between time and group were not significantly associated with HRQoL, i.e. there were no statistically significant differences between the groups over time (Time x Intervention). However, within the EQ-5D-5L, the EQ-VAS and the SF-12 PCS model, *Time* was significantly associated with HRQoL, involving a statistically significant increase of HRQoL over time within both groups (*P <0*.*05)*. The results of the final multilevel models for each HRQoL outcome are summarized in Table 2.

**Table 2:** Final multilevel model of the health-related quality of life outcomes.

	EQ-5D-5L			EQ-VAS			SF-12 PCS			SF-12 MCS		
Variable	Estimate	SE	95% CI	Estimate	SE	95% CI	Estimate	SE	95% CI	Estimate	SE	95% CI
Intercept	0.83***	0.03	0.77 − 0.89	75.70***	2.73	70.35–81.06	43.12***	1.70	39.78–46.45	44.46***	1.79	40.96–47.97
Group ^A^	0.02	0.01	-0.005 − 0.05	0.91	1.23	-1.50–3.32	2.51**	0.72	1.09–3.93	0.44	0.77	-1.07–1.95
Gender ^B^	0.04**	0.01	0.01 − 0.06	1.16	1.03	-0.86–3.27	-0.0002	0.65	-1.28–1.28	-0.43	0.68	-1.77–0.91
Age	-0.001**	0.0004	-0.002 − -0.001	-0.13**	0.04	-0.21 –-0.05	-0.02	0.03	-0.07–0.03	0.08**	0.03	0.03–0.13
Time T1	0.01*	0.01	0.002–0.03	1.32*	0.64	0.07–2.57	†	†	†	†	†	†
Time T2	0.02*	0.03	0.001–0.03	2.34*	0.92	0.53–4.16	1.39**	0.40	0.61–2.17	0.64	0.50	-0.36–1.63
Time T1 x Intervention	0.01	0.01	-0.01–0.03	0.57	0.85	-1.10–2.25	†	†	†	†	†	†
Time T2 x Intervention	0.01	0.01	-0.01 − 0.03	0.87	1.24	-1.57–3.30	-0.42	0.53	-1.47–0.63	-0.44	0.68	-1.77–0.89

^A^ Group was coded as 0 = control group and 1 = intervention group

^B^ Gender was coded as 0 = female and 1 = male; T1 = within a week after the consultation with specialist; T2 = 3 months after the consultation; SE = Standard Error; CI = Confidence interval

† = Variable not included

* P < 0.05

** P < 0.01

*** P < 0.001

### Healthcare costs

The intervention group consisted of 1,859 patients and the control group consisted of 2,045 patients. The patient characteristics and the average healthcare costs per patient at baseline, three-, six- and nine-month follow-up are presented in S3 Table and S1 Fig. The groups did significantly differ in age and gender (*p* <0.000). Table 3 summarizes the final multilevel model of the healthcare costs. With a mean difference of 107.72 euro, the average costs per patient were significantly lower at baseline within the PC+ group compared to the control group (*p <*0.001). Moreover, the interaction terms at three, six and nine months showed statistically significant results. Within the intervention group the healthcare costs increased less compared to the healthcare costs of patients in the control group (*p* <0.001). The statistical models showed that age and gender were not confounding variables, however, age and gender were significantly associated with higher healthcare costs. Older patients and males had significantly higher healthcare costs compared to younger patients and females (*p* <0.01).

**Table 3:** Final multilevel model of the healthcare costs.

	Healthcare costs		
Variable	Estimate	SE	95% CI
Intercept	471.85***	39.61	394.18–549.52
Group ^A^	-94.03***	18.20	-129.71 –-58.34
Gender ^B^	54.58**	17.75	19.78–89.37
Age (in years)	1.99**	0.58	0.84–3.13
Time T1 (3 month follow-up)	279.16***	24.19	231.73–326.60
Time T2 (6 month follow-up)	429.49***	29.95	370.78–488.21
Time T3 (9 month follow-up)	569.50***	34.98	500.91–638.09
Time T1 x Group ^A^	-182,20***	35.06	-250.95 –-113.46
Time T2 x Group ^A^	-260.25***	43.40	-345.34 –-175.16
Time T3 x Group ^A^	-364.03***	50.70	-463.43 –-264.64

Notes

^A^ Group was coded as 0 = control group and 1 = intervention group

^B^ Gender was coded as 0 = female and 1 = male; SE = Standard Error; CI = Confidence interval

** P < 0.01

*** P < 0.001

## Discussion

This study provides insight into whether the cardiology PC+ centre is able to pursue the Triple Aim [3]. In terms of patients’ satisfaction, the findings were in favour of PC+. In both groups, patients’ experience of care shows a positive picture. However, within the PC+ group, the percentage of satisfied patients was significantly higher. The findings suggest that PC+ results in improved quality of care as experienced by patients. Moreover, the findings related to the health of the study population show a statistically significant increase over time on three out of four HRQoL measurements (EQ-5D-5L, EQ-VAS and SF-12 PCS). However, the findings show no interaction effect between group and time, i.e. the increase in HRQoL was not larger within the intervention group. In conclusion, PC+ results in equal effects on HRQoL outcomes over time as compared to care-as-usual. Furthermore, the average baseline healthcare costs were significantly lower for PC+ compared to care-as-usual, and at three-, six- and nine-month follow-up the healthcare costs increased significantly less within the intervention group.

The practice-based longitudinal observational design of this study is seen as a viable alternative for the Randomized controlled trails (RCT) design [33, 34]. RCTs are assumed to be stronger on internal validity, however, difficult to perform in practice. Thereby, the external validity of practice-based research is commonly higher compared to the RCT design. The findings of practice-based observational research are more generalizable and can be (directly) translated into practice [34]. Moreover, the use of MLM and gain score analyses strengthens this study, since it accounts for dependency of observations in time and the group effect is adjusted for the complete group difference in the baseline measurement [31, 35]. Compared with the ANCOVA technique, GSA yields less biased results in non-randomized controlled studies where ‘natural’ groups are compared which is the case in this practice-based observational study with non-randomized groups. The ANCOVA gives a smaller standard error of the estimated treatment effect compared to GSA, meaning that the GSA has a larger power [31, 32].

A limitation of this study is the sample size of the control group within the questionnaire study. Due to practical and privacy regulation issues, it was hard to set up a straightforward procedure for the inclusion of patients within the questionnaire study. This resulted in a response rate of 20.2% in the intervention group and a response rate of 10.7% in the control group. A sample size of 358 patients was required according to the power calculation; this was not achieved in the control group; only 321 patients were included after more than two years of inclusion. Furthermore, HRQoL was measured with the generic instruments EQ-5D-5L and SF-12. Generic HRQoL instruments are limited in their responsiveness and ability to discriminate between health states [36]. Condition-specific instruments are likely to be more responsive. Using condition-specific instruments, focused on health aspects that are specifically important for a specific patient group, could have resulted in more statistically significant findings [36]. However, generic instruments are applicable across conditions and interventions and make it possible to compare results across interventions. This was one of the main reasons for choosing generic questionnaires within this study. Future research will focus on other medical specialties and other appearances of PC+ interventions and it would be interesting to compare the results on HRQoL (and also on patients’ experience of care and healthcare costs).

The importance of patients’ knowledge on health and health care as a source of improving the quality of care is increasingly recognized [26, 37, 38]. Since there is an emerging consensus that patients’ experiences are a fundamental aspect of quality of care, the perspective of patients is more and more integrated in the evaluation of (new) healthcare delivery models [3, 39, 40]. Previous research on PC+ and other substitution initiatives such as joint consultations and outreach specialist services show similar results regarding patient satisfaction with quality of care [14, 17, 41]. Moreover, the literature did not show any consistent evidence about the impact of substitution initiatives on health outcomes [18, 19, 41, 42]. Hence, more research, and probably condition-specific instruments, are needed to investigate the impact of substitution initiatives and in particular of PC+ on health outcomes.

Furthermore, although the results of this study show lower healthcare costs at six and nine months’ follow-up for patients referred to PC+, other studies show that substitution initiatives could also lead to increased healthcare costs caused by the inefficient use of medical specialists’ time, travel time (and costs), overhead costs and staffing costs [17, 41, 43]. This study focused on the healthcare costs claimed at micro level (i.e. patient level). Before implementation on a larger scale, the longitudinal effects of PC+ should be investigated at macro level (i.e. regional and national level). Additionally, this study did only take into account the healthcare costs related to cardiology-related healthcare services provided in the PC+ centre and the hospital. It would be interesting to investigate the effect of PC+ on the healthcare costs from a broader perspective by for example also taking into consideration primary care, social care and drugs.

To optimize the healthcare system by successfully achieving improvements on the Triple Aim, healthcare organizations should be highly effective [44, 45]. Whether a healthcare organization is highly effective depends largely on the performance of the healthcare providers. This means that the sustainability of new healthcare delivery models, such as PC+, is determined by the support and performance of healthcare providers. Consequently, it is recommended to include the fourth aim ‘improving the experience of providing care’ and change the Triple Aim into the Quadruple Aim [44, 45]. Hence, the experience of healthcare providers (e.g. GPs and medical specialists) with PC+ should be examined.

In the coming years research will also focus on other medical specialties within a PC+ setting (e.g. internal medicine, dermatology, minor surgical operation and ear, nose and throat care) and on comparing (results of) different appearances of PC+ interventions in other Dutch regions.

## Conclusion

Overall, PC+ seems to be a promising new healthcare delivery model as it could lead to improved patients’ experience of care, equal HRQoL outcomes and reduced healthcare costs per patient as compared to care-as-usual.

## Supporting information

S1 File Questions related to the items in Table 1; measuring the patients’ experience of care. (DOCX)

S1 Table Patient characteristics and descriptive statistics concerning the patient questionnaire study (measuring patients’ experience of care and HRQoL). (DOCX)

S2 Table Multilevel models of the health-related quality of life outcomes. (DOCX)

S3 Table Patient characteristics and descriptive statistics regarding the evaluation of the healthcare costs. (DOCX)

S1 Fig Visualization of the average healthcare costs per patient: Intervention group versus control group. Notes: * groups differ statistically significantly with a P-value < 0.05.
(DOCX)

S1 Data Patient questionnaire data. (XLSX)

S2 Data Administrative healthcare data extracted from the electronic medical record system of the PC+ centre and the hospital. (XLSX)

## Abbreviations

CI: Confidence interval
EQ-5D-5L: EuroQol, five-dimensional questionnaire five-level version
EQ-VAS: EuroQol Visual Analogue Scale
GP: General Practitioner
GSA: Gain score analysis
HBOC: Hospital-based outpatient care
HRQoL: Health-related quality of life
MCS: Mental component summary
MLM: Multilevel linear modelling
PC+: Primary care plus
PCS: Physical component summary
RCT: Randomized controlled trial
SE: Standard error
SF-12: Short-Form Health Survey
WHO: World Health Organization
