# Supplementary material for: Substituting hospital-based outpatient cardiology care: The impact on quality, health and costs
Source: PLoS One. 2019 May 31;14(5):e0217923. doi: 10.1371/journal.pone.0217923 (PMC6544378; doi:10.1371/journal.pone.0217923)
Supplement: S1 File — (DOCX) [file pone.0217923.s001.docx]

**Questions related to the items in table 1; measuring the patients’ experience of care.**

**General items related to the experience of care**

1. Are you satisfied with the time that you could get an appointment?

- Satisfaction with waiting time for appointment

Response options: not at all; a little bit; largely; totally

1. Did you find PlusPunt/the hospital easily?

- Findability of location

Response options: not at all; a little bit; largely; totally

1. Did you feel welcome and comfortable at the PC+ centre/the hospital?

- Feeling welcome and comfortable

Response options: not at all; a little bit; largely; totally

1. Did the healthcare assistant help you well?

- Helpful healthcare assistant

Response options: not at all; a little bit; largely; totally

1. Did the healthcare assistant explain everything in an understandable way?

- Understandable explanation by healthcare assistant

Response options: not at all; a little bit; largely; totally

1. Were there sufficient facilities in the waiting room (e.g. magazines, something to drink, toys, and brochures)?

- Sufficient facilities in waiting room

Response options: not at all; a little bit; largely; totally

1. Were the health professionals were informed about the complaint for which you were referred by the general practitioner?

- Healthcare professionals were informed about the complaint

Response options: not at all; a little bit; largely; totally

1. Was your complaint taken seriously?

- Complaint was taken seriously

Response options: not at all; a little bit; largely; totally

1. Did the healthcare professionals listen carefully to you?

- Healthcare professionals listened carefully

Response options: not at all; a little bit; largely; totally

1. Did the healthcare professionals take enough time for you?

- Healthcare professionals took enough time

Response options: not at all; a little bit; largely; totally

1. Did the healthcare professionals treat you with respect?

- Healthcare professionals treated you with respect

Response options: not at all; a little bit; largely; totally

1. Were the healthcare professionals competent?

- Competence of healthcare professionals

Response options: not at all; a little bit; largely; totally

1. Are you satisfied with the help of the healthcare professionals?

- Overall help of healthcare professionals

Response options: not at all; a little bit; largely; totally

1. Did the healthcare professionals explain everything in an understandable way?

- Understandable explanation of healthcare professionals

Response options: not at all; a little bit; largely; totally

1. Did you get the opportunity to ask all your questions?

- Opportunity to ask questions

Response options: not at all; a little bit; largely; totally

1. How would you assess the collaboration and alignment between the GP and the PC+ centre/the hospital in general?

- Collaboration and alignment of care with GP

Response options: excellent; good; moderate; bad

**Specific items related to the experience of care with the medical specialist (i.e. the cardiologist)**

1. Did the medical specialist take enough time for you?

- The medical specialist took enough time

Response options: not at all; a little bit; largely; totally

1. Was the medical specialist informed about the complaint for which you were referred by the general practitioner?

- The medical specialist was informed about complaint

Response options: not at all; a little bit; largely; totally

1. Did the medical specialist give sufficient and understandable explanation about the results of the consultation and diagnostic tests?

- The medical specialist explained the results of the consultation and diagnostics sufficiently and understandable

Response options: not at all; a little bit; largely; totally

1. Did the medical specialist provide information about the different treatment options for your health complaint?

- The medical specialist provide information about the treatment options

Response options: not at all; a little bit; largely; totally

1. Were you involved in the decision about the treatment or assistance you got?

- Involved the patient in decision about the treatment

Response options: not at all; a little bit; largely; totally

**Additional questions**

1. What was the waiting time before you could get an appointment?

- Waiting time for appointment

Response options: within than 3 days; within 7 days; between 8 and 14 days; more than 14 days

1. How long did you have to wait in the waiting room after the agreed time?

- Waiting time in waiting room

Response options: less than 15 minutes; between 15 and 30 minutes; between 30 and 60 minutes; more than 60 minutes

1. Would you recommend the PC+ centre/ the hospital to your friends and family?

- Recommendation of PC+ centre/hospital

Response options: not at all; a little bit; largely; totally

1. Would you recommend the medical specialist to your friends and family?

- Recommendation of medical specialist

Response options: not at all; a little bit; largely; totally

1. What grade between 0 and 10 would you give the PC+/the hospital?

- Grade for PC+/hospital

Response options: a number between 0 and 10

1. What grade between 0 and 10 would you give the medical specialist?

- Grade for PC+/hospital

Response options: a number between 0 and 10
