# Supplementary material for: Substituting hospital-based outpatient cardiology care: The impact on quality, health and costs
Source: PLoS One. 2019 May 31;14(5):e0217923. doi: 10.1371/journal.pone.0217923 (PMC6544378; doi:10.1371/journal.pone.0217923)
Supplement: S1 Table — (DOCX) [file pone.0217923.s002.docx]

**Appendix 2a: Patient characteristics and descriptive statistics concerning the patient questionnaire study (measuring patients’ experience of care and HRQoL).**

|  | **Intervention group (PC+)** | **Control group (HBOC)** | ***P*-value** |
| --- | --- | --- | --- |
| **Age in years** mean (±SD) | 57.28 (±13.24) | 63.89 (±12.94) | < 0.000 * |
| **Gender** % (n)  Male  Female | 47.71 % (208) 52.29 % (228) | 51.23 % (167)  48.77 % (159) | 0.336 |
| **EQ-5D-5L** mean (±SD)  Baseline  T1  T2 | 0.78 (±0.16)  0.80 (±0.15)  0.81 (±0.17) | 0.75 (±0.18)  0.77 (±0.18)  0.78 (±0.18) | 0.025 *  0.008 *  0.052 * |
| **EQ-VAS** mean (±SD)  Baseline  T1  T2 | 69.91 (±15.58)  71.87 (±14.84)  73.27 (±14.74) | 68.06 (±17.15)  69.67 (±15.94)  70.76 (±16.86) | 0.131  0.071  0.061 |
| **SF-12 PCS** mean (±SD)  Baseline  T2 | 44.56 (±9.08)  45.66 (±8.65) | 41.84 (±10.06)  43.66 (±9.59) | < 0.000 *  0.008 * |
| **SF-12 MCS** mean (±SD)  Baseline  T2 | 49.15 (±10.36)  49.96 (±9.56) | 49.18 (±9.96)  50.16 (±10.17) | 0.964  0.805 |

Notes: *T1 = within a week after the consultation; T2 = 3 months after the consultation; n= number of participants who filled in the questionnaire; SD = Standard deviation;* * *groups differ significantly with a p-value < 0.05*
