# Supplementary material for: Substituting hospital-based outpatient cardiology care: The impact on quality, health and costs
Source: PLoS One. 2019 May 31;14(5):e0217923. doi: 10.1371/journal.pone.0217923 (PMC6544378; doi:10.1371/journal.pone.0217923)
Supplement: S2 Table — (DOCX) [file pone.0217923.s003.docx]

**Apendix 2b Multilevel models of the health-related quality of life outcomes.**

|  | |  | **Model 1** | | | **Model 2** | | | **Final model** | | |
| --- | --- | --- | --- | --- | --- | --- | --- | --- | --- | --- | --- |
|  | |  | Estimate | SE | 95% CI | Estimate | SE | 95% CI | Estimate | SE | 95% CI |
| **EQ-5D-5L** | Intercept | | 0.75*** | 0.01 | 0.73 – 0.77 | 0.84*** | 0.03 | 0.78 – 0.90 | 0.83*** | 0.03 | 0.77 − 0.89 |
|  | Group ^A^ | | 0.03* | 0.01 | 0.005 – 0.05 | 0.02 | 0.01 | -0.01 – 0.05 | 0.02 | 0.01 | -0.005 − 0.05 |
|  | Gender ^B^ | |  |  |  |  |  |  | 0.04** | 0.01 | 0.01 − 0.06 |
|  | Age | |  |  |  | -0.001** | 0.0004 | -0.002 – -0.0005 | -0.001** | 0.0004 | -0.002 − -0.001 |
|  | Time T1 | | 0.01* | 0.01 | 0.002 – 0.03 | 0.01* | 0.01 | 0.002 – 0.03 | 0.01* | 0.01 | 0.002 – 0.03 |
|  | Time T2 | | 0.02* | 0.01 | 0.001 – 0.03 | 0.02* | 0.01 | 0.001 – 0.03 | 0.02* | 0.03 | 0.001 – 0.03 |
|  | Time T1 x Intervention | | 0.01 | 0.01 | 0.005 – 0.03 | 0.01 | 0.01 | -0.01 – 0.03 | 0.01 | 0.01 | -0.01 – 0.03 |
|  | Time T2 x Intervention | | 0.005 | 0.01 | -0.02 – 0.03 | 0.01 | 0.01 | -0.02 – 0.03 | 0.01 | 0.01 | -0.01 − 0.03 |
| **EQ-VAS** | Intercept | | 68.01*** | 0.91 | 66.22 – 69.80 | 76.11*** | 2.70 | 70.81 – 81.42 | 75.70*** | 2.73 | 70.35 – 81.06 |
|  | Group ^A^ | | 1.74 | 1.20 | -0.62 – 4.10 | 0.88 | 1.23 | -1.53 – 3.29 | 0.91 | 1.23 | -1.50 – 3.32 |
|  | Gender ^B^ | |  |  |  |  |  |  | 1.16 | 1.03 | -0.86 – 3.27 |
|  | Age | |  |  |  | -0.13** | 0.4 | -0.21 – -0.05 | -0.13** | 0.04 | -0.21 – -0.05 |
|  | Time T1 | | 1.33* | 0.64 | 0.08 – 2.58 | 1.33* | 0.64 | 0.08 – 2.58 | 1.32* | 0.64 | 0.07 – 2.57 |
|  | Time T2 | | 2.33* | 0.92 | 0.51 – 4.14 | 2.37* | 0.92 | 0.55 – 4.18 | 2.34* | 0.92 | 0.53 – 4.16 |
|  | Time T1 x Intervention | | 0.51 | 0.85 | -1.16 – 2.18 | 0.56 | 0.85 | -1.11 – 2.23 | 0.57 | 0.85 | -1.10 – 2.25 |
|  | Time T2 x Intervention | | 0.71 | 1.24 | -1.72 – 3.15 | 0.85 | 1.24 | -1.59 – 3.28 | 0.87 | 1.24 | -1.57 – 3.30 |
| **SF-12 PCS** | Intercept | | 41.85*** | 0.53 | 40.81 | 43.12*** | 1.70 | 39.78 – 46.45 | 43.12*** | 1.70 | 39.78 – 46.45 |
|  | Group ^A^ | | 2.65*** | 0.70 | 1.27 – 4.03 | 2.51** | 0.722 | 1.10 – 3.93 | 2.51** | 0.72 | 1.09 – 3.93 |
|  | Gender ^B^ | |  |  |  |  |  |  | -0.0002 | 0.65 | -1.28 –1.28 |
|  | Age | |  |  |  | -0.02 | 0.03 | -0.07 – 0.03 | -0.02 | 0.03 | -0.07 – 0.03 |
|  | Time T2 | | 1.39** | 0.40 | 0.61 – 2.17 | 1.39** | 0.40 | 0.61 – 2.17 | 1.39** | 0.40 | 0.61 –2.17 |
|  | Time T2 x Intervention | | -0.42 | 0.53 | -1.47 – 0.62 | -0.12 | 0.53 | -1.47 – 0.63 | -0.42 | 0.53 | -1.47 – 0.63 |
| **SF-12 MCS** | Intercept | | 43.23*** | 0.57 | 48.11 – 50.34 | 44.30*** | 1.77 | 40.83 – 47.77 | 44.46*** | 1.79 | 40.96 – 47.97 |
|  | Group ^A^ | | -0.08 | 0.75 | -1.55 – 1.40 | 0.45 | 0.77 | -1.06 – 1.96 | 0.44 | 0.77 | -1.07 – 1.95 |
|  | Gender ^B^ | |  |  |  |  |  |  | -0.43 | 0.68 | -1.77 – 0.91 |
|  | Age | |  |  |  | 0.08** | 0.03 | 0.03 – 0.13 | 0.08** | 0.03 | 0.03 – 0.13 |
|  | Time T2 | | 0.63 | 0.50 | -0.36 – 1.62 | 0.63 | 0.50 | -.036 – 1.63 | 0.64 | 0.50 | -0.36 – 1.63 |
|  | Time T2 x Intervention | | -0.39 | 0.68 | -1.72 – 0.94 | -0.43 | 0.68 | -1.77 – 0.90 | -0.44 | 0.68 | -1.77 – 0.89 |

*Notes: ^A^ Group was coded as 0=control group and 1=intervention group; ^B^ Gender was coded as 0=female and 1=male 0; T1 = within a week after the consultation; T2 = 3 months after the consultation; SE = Standard Error; CI = Confidence interval; † = Variable not included; * groups differ significantly with a p <0.05; ** groups differ significantly with a p < 0.01; *** groups differ significantly with a p < 0.001*
