# Supplementary material for: Substituting hospital-based outpatient cardiology care: The impact on quality, health and costs
Source: PLoS One. 2019 May 31;14(5):e0217923. doi: 10.1371/journal.pone.0217923 (PMC6544378; doi:10.1371/journal.pone.0217923)
Supplement: S3 Table — (DOCX) [file pone.0217923.s004.docx]

**Appendix 3a: Patient characteristics and descriptive statistics regarding the evaluation of the healthcare costs**

|  | **Intervention group (PC+)** | **Control group (HBOC)** | ***P*-value** |
| --- | --- | --- | --- |
| **Age in years** mean (±SD) | 57.12 (±14.22) | 62.22 (±15.95) | <0.000 |
| **Gender** % (n)  Male  Female | 42.50% (790)  57.50% (1069) | 48.00% (1002)  51.00% (1043) | <0.000 |
| **Average healthcare costs**  mean (±SD)  Baseline    3 month follow-up    6 month follow-up    9 month follow-up | 514.74 (±0.000)  611.70 (±1095.73)  683.99 (±1258.32)  720.21 (±1334.39) | 622.46 (±775.40)  901.63 (±1466.44)  1051.96 (±1778.00)  1191.96 (±2086.06) | <0.000  <0.000  <0.000  <0.000 |
