# Supplementary material for: Substituting hospital-based outpatient cardiology care: The impact on quality, health and costs
Source: PLoS One. 2019 May 31;14(5):e0217923. doi: 10.1371/journal.pone.0217923 (PMC6544378; doi:10.1371/journal.pone.0217923)

**Appendix 3b: Visualization of the average healthcare costs per patient: intervention group versus control group; *Notes: * groups differ statistically significant with a P-value < 0.05.***


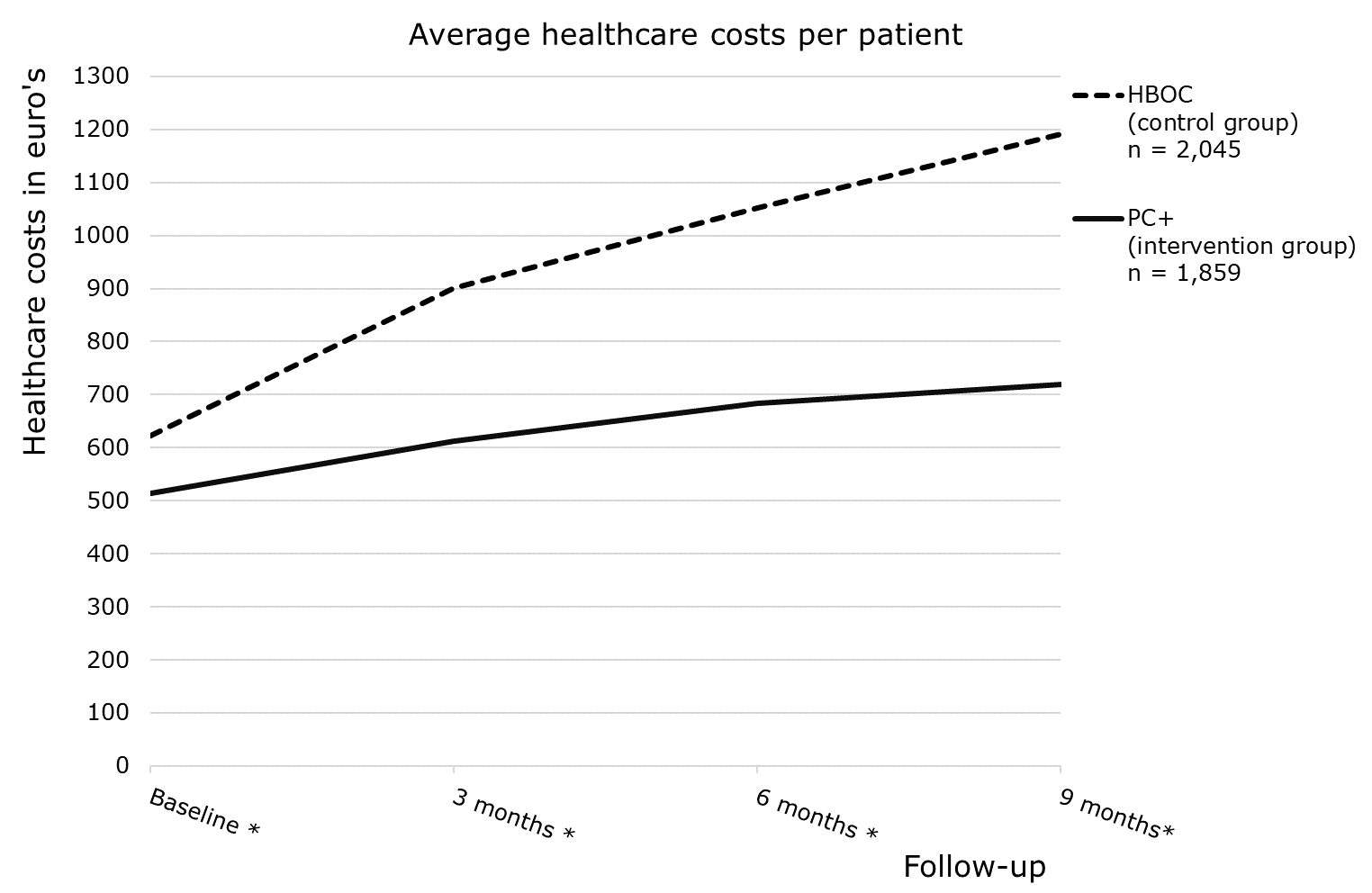

Supplement: S1 Fig — Notes: * groups differ statistically significantly with a P-value < 0.05. (DOCX) [file pone.0217923.s005.docx]
